# Emerging toolkits for decoding the co-occurrence of modified histones and chromatin proteins

**DOI:** 10.1038/s44319-024-00199-2

**Published:** 2024-08-02

**Authors:** Anne-Sophie Pepin, Robert Schneider

**Affiliations:** 1 Institute of Functional Epigenetics (IFE), Helmholtz Zentrum München, Neuherberg, Germany; 2 Faculty of Biology, Ludwig-Maximilians-Universität München, Planegg-Martinsried, Germany

**Keywords:** Single-cell Omics, Histone Posttranslational Modifications, Nonhistone Chromatin Proteins, Combinatorial Occurrence, Chromatin States, Chromatin, Transcription & Genomics, Methods & Resources

## Abstract

In eukaryotes, DNA is packaged into chromatin with the help of highly conserved histone proteins. Together with DNA-binding proteins, posttranslational modifications (PTMs) on these histones play crucial roles in regulating genome function, cell fate determination, inheritance of acquired traits, cellular states, and diseases. While most studies have focused on individual DNA-binding proteins, chromatin proteins, or histone PTMs in bulk cell populations, such chromatin features co-occur and potentially act cooperatively to accomplish specific functions in a given cell. This review discusses state-of-the-art techniques for the simultaneous profiling of multiple chromatin features in low-input samples and single cells, focusing on histone PTMs, DNA-binding, and chromatin proteins. We cover the origins of the currently available toolkits, compare and contrast their characteristic features, and discuss challenges and perspectives for future applications. Studying the co-occurrence of histone PTMs, DNA-binding proteins, and chromatin proteins in single cells will be central for a better understanding of the biological relevance of combinatorial chromatin features, their impact on genomic output, and cellular heterogeneity.

## Abstract (web summary)

This review discusses state-of-the-art techniques for the simultaneous profiling of multiple chromatin features in low-input samples or single cells, focusing on histone modifications, DNA-binding and chromatin proteins.

## Graphical abstract

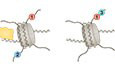

## Introduction

Regulation of gene expression involves the complex and coordinated integration of numerous factors including chromatin architecture, regulatory elements, histone, RNA and DNA modifications, as well as proteins interacting with DNA and/or chromatin. Eukaryotic DNA is packaged into nucleosomes whereby 147 bases of DNA are wrapped around a histone protein octamer containing two copies each of histone H2A, H2B, H3, and H4 (Luger et al, 1997). Histones carry many posttranslational modifications (PTMs), both within their core and also on the tails protruding from the nucleosome (Turner, 1993, 2000; Strahl and Allis, 2000; Jenuwein and Allis, 2001; Zhao and Garcia, 2015). Advances in proteomic technologies and chromatin research have enabled the identification of novel histone PTMs, with currently a growing catalog of more than 25 different types of PTMs and over a hundred distinct possible sites for modifications, considerably extending the so-called “histone code” (Turner, 1993, 2000; Strahl and Allis, 2000; Jenuwein and Allis, 2001; Zhao and Garcia, 2015; Millán-Zambrano et al, 2022). These modifications can affect chromatin structure directly or determine the binding of effector proteins (readers) that can facilitate DNA-templated processes (Millán-Zambrano et al, 2022; Lukauskas et al, 2024; Policarpi et al, 2024). Importantly, mass spectrometry-based methods and sequential chromatin immunoprecipitation (ChIP) experiments have identified combinatorial occurrence (or co-occurrence) of histone PTMs, giving rise to a vast space for combinatorial modification states that could alter, for example, binding affinities for reader proteins (Bernstein et al, 2006; Sidoli et al, 2012; Voigt et al, 2012; Schwämmle et al, 2014; Bai et al, 2018; Janssen et al, 2019; Lu et al, 2021). Of note, histone PTMs are only one example out of many chromatin components that can display co-occurrence, as also DNA- and chromatin-binding proteins, as well as modifications of DNA, can co-occur and potentially act in concert.

**Figure 1:**
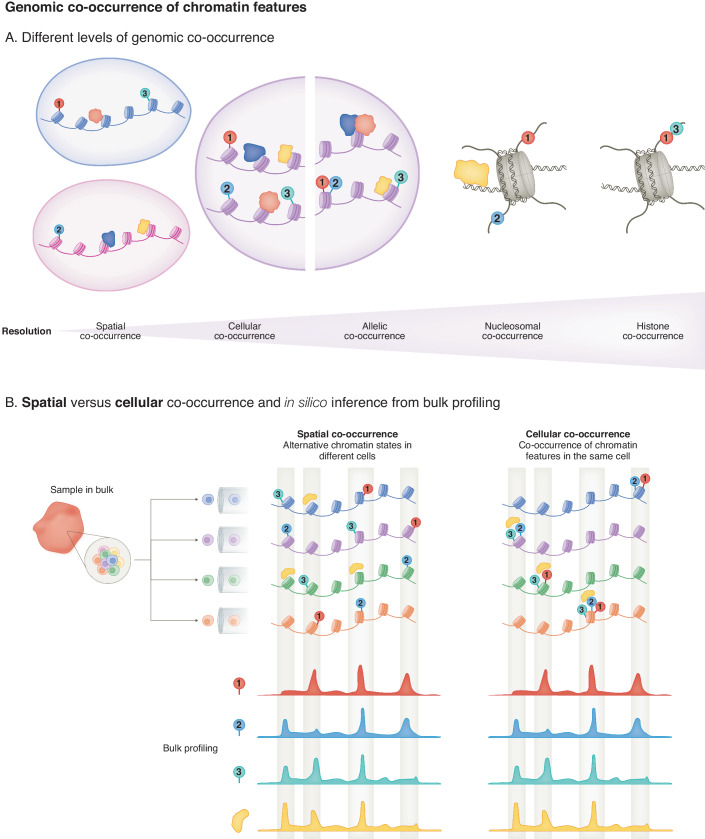
Genomic co-occurrence of chromatin features. (**A**) Different levels of genomic co-occurrence. Spatial co-occurrence refers to the combinatorial presence of multiple chromatin features at the same genomic coordinates irrespective of whether this occurs within the same cell. Co-occurrence can also take place within the same cell (cellular co-occurence), on different alleles or on the same allele (allelic co-occurrence). Co-occurrence can occur within a single nucleosome (nucleosomal co-occurrence), and for histone PTMs either on different histone proteins in the same nucleosome or on the same histone protein (histone co-occurrence). (**B**) Spatial versus cellular co-occurrence and in silico inference from bulk profiling. Schematic representation of spatial co-occurrence where different chromatin features are found at the same genomic sites but in different cells (upper left panel), versus cellular co-occurrence where multiple chromatin features are found at a specific locus within the same single cell (upper right panel, with sites of cellular co-occurrence highlighted in dark blue). Note the corresponding enrichment signals derived from bulk experiments (lower panel).

In general, the concept of genomic co-occurrence as we use it here, involves the presence of multiple chromatin components. The co-occurrence of histone PTMs, DNA-binding proteins, and/or chromatin-binding proteins is the focus of this review, referred to as “chromatin features”, and is thought to provide functional synergy, and enable the fine-tuning of transcriptional outputs (Goudarzi et al, 2016; Gao et al, 2021; Policarpi et al, 2024). Importantly, the level of genomic co-occurrence that can be detected by specific approaches is dependent on their resolution, and determines which biological questions can be addressed (see Fig. 1A). Specifically, we define **spatial** co-occurrence as co-occurring histone PTMs or co-occupying proteins at the same genomic coordinates, irrespective of whether this occurs within the same cell. In contrast, **cellular** co-occurrence indicates co-occurrence within the same cell. Cellular co-occurrence may take place on two different alleles, or within the same allele (**allelic** co-occurrence). Co-occurrence can also occur within a single nucleosome (**nucleosomal** co-occurrence). Lastly, multiple histone PTMs may be present within a single nucleosome either on two different copies of a histone protein, or on the same histone protein (**histone** co-occurrence). Overall, these different levels of genomic co-occurrence can reflect important information on the functional cooperativity of chromatin features that may result in varying transcriptional outputs within a complex biological context. However, current challenges to study the different levels of co-occurrence high-throughput or genome-wide limit our understanding of the functional relevances of the co-occurrences of chromatin features (Voigt et al, 2012; Sidoli and Garcia, 2015).

Methods to map the genome-wide distribution of DNA-binding proteins, chromatin proteins, or histone PTMs have provided valuable insights into gene regulatory networks. Thus far, such approaches have largely focused on profiling individual targets in bulk samples. Integration of multiple datasets from bulk genome-wide mapping studies can provide hints on potential sites of **spatial** co-occurrence by identifying genomic regions where chromatin features are enriched in distinct datasets (Fig. 1B). Importantly, collaborative efforts such as the ENCODE project have built a useful resource to obtain datasets of an increasing number of targets, from cell lines and tissues, from different source organisms, generated with standardized procedures, that can serve for in silico analysis of genomic co-occurrence of chromatin factors (Dunham et al, 2012; Akbarian et al, 2015; Abascal et al, 2020; Partridge et al, 2020). However, the co-occurrence inferred from such in silico comparative analysis cannot distinguish between overlapping chromatin features that are due to alternative binding events in different population of cells (spatial co-occurrence) rather than “*true”* co-occurrence within the same cell (cellular co-occurrence; Fig. 1B).

To obtain a better understanding of the cooperative function of chromatin features, methods that can simultaneously profile the **cellular** co-occurrence of multiple histone PTMs and nonhistone proteins are required. Generating precise chromatin state maps with single-cell resolution will allow to unambiguously study the multifactorial nature of gene expression regulation. The past decade has been marked by a growing toolkit of newly developed single-cell and spatial multi-omics methodologies, and some of these methods have been recently reviewed in (Vandereyken et al, 2023). It is only within the last 3 years that ground-breaking single-cell -omics advances now permit to profile the cellular co-occurrence of multiple chromatin features. In this context, we cover the underlying methodological principles and provide perspectives on advances to allow for the simultaneous multi-mapping of chromatin features in low-input samples and in particular in single cells. We compare and contrast the toolkits that are based on ChIP, as well as those involving enzyme-tethering approaches: targeted DNA adenine methylation tagging, targeted chromatin cleavage, and antibody-tethered tagmentation. We further elaborate on the limitations and challenges associated with these different approaches for profiling the co-occurrence of chromatin features, and the considerations to account for based on the characteristic features of the currently established toolkits. Finally, we provide perspectives on the challenges and limitations that still have to be overcome moving forward. This review provides background on the underlying principles of such approaches and can help researchers in the planning of future experiments to determine the most appropriate method to pursue for multifactorial profiling in a complex biological context.

## Section 1: ChIP-based methods and related approaches

**Figure 2:**
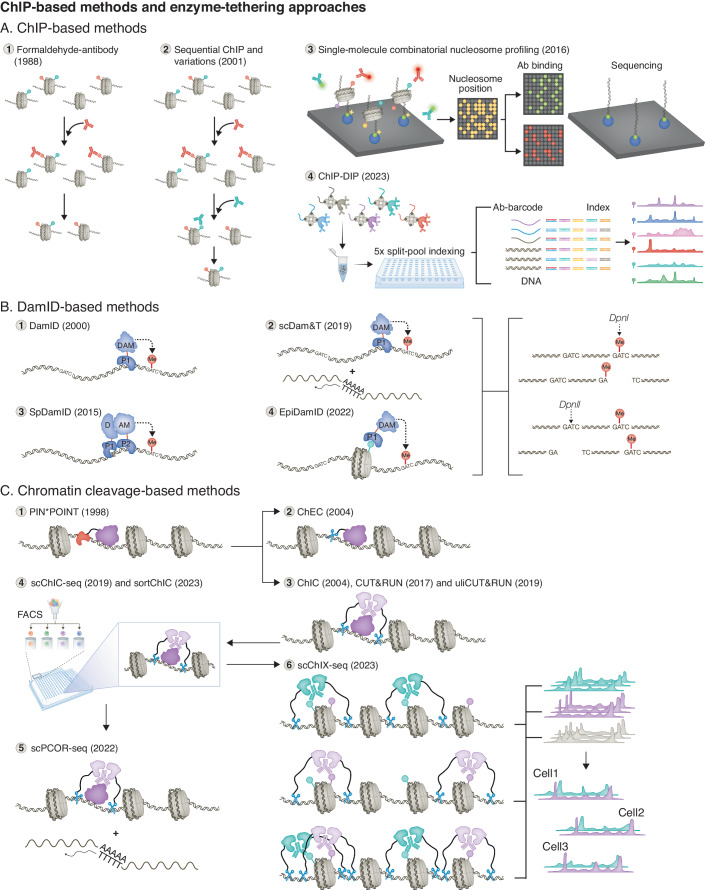
ChIP-based methods and enzyme-tethering approaches. Overview of ChIP-based methods (**A**), targeted DNA adenine methylation-based approaches (**B**), and targeted DNA cleavage-based techniques (**C**), to profile genomic sites of co-occurring chromatin features. For further details on the methods, see main text. (**A)** ChIP-based methods. (1) Formaldehyde-antibody method (Hebbes et al, 1988; Solomon et al, 1988). Chromatin fragments are subjected to immunoprecipitation using antibodies specific for a protein of interest. (2) Sequential ChIP and variations (Chaya et al, 2001). Chromatin fragments are subjected to a first immunoprecipitation using antibodies specific to a chromatin feature of interest, followed by a second immunoprecipitation step targeting another feature to enrich for fragments with both features. (3) Single-molecule combinatorial nucleosome profiling (Shema et al, 2016). Chromatin is digested by MNase and mononucleosomes are ligated to fluorescent adapters and captured on slides. Total internal reflection (TIRF) microscopy is used to image nucleosome positions. Following the release of the fluorophores, fluorescently labeled antibodies targeting histone PTMs are applied to the slides and antibody binding events are recorded. DNA sequence is determined by NGS. (4) ChIP-DIP (preprint: Perez et al, 2023). Streptavidin beads are bound with biotinylated antibody-specific barcoded adapters, and with biotinylated protein G beads bound with antibodies, to create a bead pool. Following crosslinking and cell lysis, the bead pool is applied to perform ChIP. A total of five rounds of split-pool indexing can assign the genomic DNA sequences to their associated antibodies. (**B)** DamID-based methods. (1) DamID (Steensel and Henikoff 2000). Dam is tethered by its fusion partner to the genomic binding sites of a protein of interest (P1) and methylates adenines within nearby GATC motifs. (2) scDam&T (Rooijers et al, 2019). An additional reverse transcription step allows to jointly measure gene expression and profile a chromatin protein in single cells. (3) SpDamID (Hass et al, 2015). Two fusion proteins are generated, consisting of two nonhistone chromatin proteins (P1 and P2) and two halves of Dam (D + AM). Upon the reconstitution of two halves of Dam, Dam methylates adenines in GATC motifs in the vicinity of the binding sites. (4) EpiDamID (Rang et al, 2022). A fusion protein consisting of Dam and a chromatin reader domain is used to tether Dam near genomic sites associated with a histone PTM of interest. (**C**) Chromatin cleavage-based methods. (1) PIN*POINT (Lee et al, 1998). A fusion protein consisting of a protein of interest (POI) and a nuclease is used to tether the cleavage of DNA at the genomic target sites of the POI. (2) ChEC (Schmid et al, 2004). A fusion protein composed of a POI and a micrococcal nuclease (MNase) is generated to direct DNA cleavage at the POI binding sites in the genome. (3) ChIC, CUT&RUN and uliCUT&RUN (Schmid et al, 2004; Skene and Henikoff, 2017; Hainer et al, 2019). Samples are successively treated with antibodies targeting a chromatin protein of interest and a protein A and MNase (pA-MNase) fusion that will cleave and release DNA fragments bound by the targeted protein. (4) scChIC-seq and sortChIC (Ku et al, 2019; Zeller et al, 2023). Cells are treated with antibodies and pA-MNase and then single cells are distributed on a plate using fluorescence-activated cell sorting (FACS). ChIC is performed in each individual well. (5) scPCOR-seq (Pan et al, 2022). In addition to the scChIC-seq/sortChIC workflow, a reverse transcription step captures simultaneously mRNAs. (6) scChIX-seq (Yeung et al, 2023). Three separate sortChIC maps are generated by using two distinct antibodies separately and by using both antibodies simultaneously. The two first maps are used to assign the fragments to their respective target.

Early methodological milestones attempting to map DNA-protein contacts date 40 years back, with UV crosslinking-immunoprecipitation methods, formaldehyde-pronase techniques, and formaldehyde-antibody approaches (Fig. 2A-1) (Gilmour and Lis, 1984, 1985, 1986; Solomon and Varshavsky, 1985; Hebbes et al, 1988; Solomon et al, 1988). Since then, ChIP has been widely used to map the binding profiles of DNA-binding proteins, chromatin proteins and histone PTMs in vivo and has been for many years the gold standard method for this purpose. ChIP can be performed with crosslinking to preserve protein–DNA interactions (X-ChIP) or alternatively without a crosslinking step under native conditions (N-ChIP). Subsequently, the chromatin is fragmented by sonication or enzymatic digestion (Skene and Henikoff, 2015). The sheared chromatin is then incubated with an antibody specific to a protein or histone PTM of interest to immunoprecipitate and enrich for the targeted proteins together with the DNA fragments they are binding to. Next, crosslinks are reversed, and the bound DNA fragments are purified. To determine the sequences enriched with a specific protein following the ChIP, hybridization assays (Hebbes et al, 1988; Dedon et al, 1991; Orlando et al, 1997), polymerase chain reaction (PCR) with primers specific to genomic regions (ChIP-qPCR), and microarray hybridization as a global mapping strategy (ChIP-chip, (Ren et al, 2000; Boyer et al, 2005; Kim et al, 2005a, 2005b; Lee et al, 2006), can be used. ChIP combined with next-generation sequencing (NGS; ChIP-seq) allows now to assay the enrichment of proteins genome-wide with base-pair resolution (Barski et al, 2007; Mikkelsen et al, 2007; Park, 2009; Furey, 2012).

Several variations of ChIP have been developed to assess whether two proteins (or histone PTMs) associate with the same genomic binding site in vivo, such as sequential ChIP (seq-ChIP), reChIP, co-ChIP, double ChIP, chromatin double immunoprecipitation (ChDIP), and others (Fig. 2A-2) (Chaya et al, 2001; Geisberg and Struhl, 2004; Henry et al, 2003; IJpenberg et al, 2004; Métivier et al, 2003; Proft and Struhl, 2002; Scully et al, 2000; Soutoglou and Talianidis, 2002). Using mononucleosomes as input, these methods involve two sequential rounds of immunoprecipitation: a first immunoprecipitation of protein–DNA complexes with a first antibody, followed by a second one with an antibody against a different target, thereby enriching for DNA bound by both targets. These different sequential ChIP variations allow to determine allelic or nucleosomal co-occurrence genome-wide (Kinkley et al, 2016; Weiner et al, 2016). While ChIP-seq-based assays are amenable to ultra-low-input samples and single-cell profiling (Brind’Amour et al, 2015; Rotem et al, 2015; Grosselin et al, 2019), and reChIP-seq has been recently adapted for low-input samples (Seneviratne et al, 2024), to our knowledge, further optimizations would have to be achieved to apply seq-ChIP, reChIP-seq, or co-ChIP-seq, in single cells.

**Figure 3:**
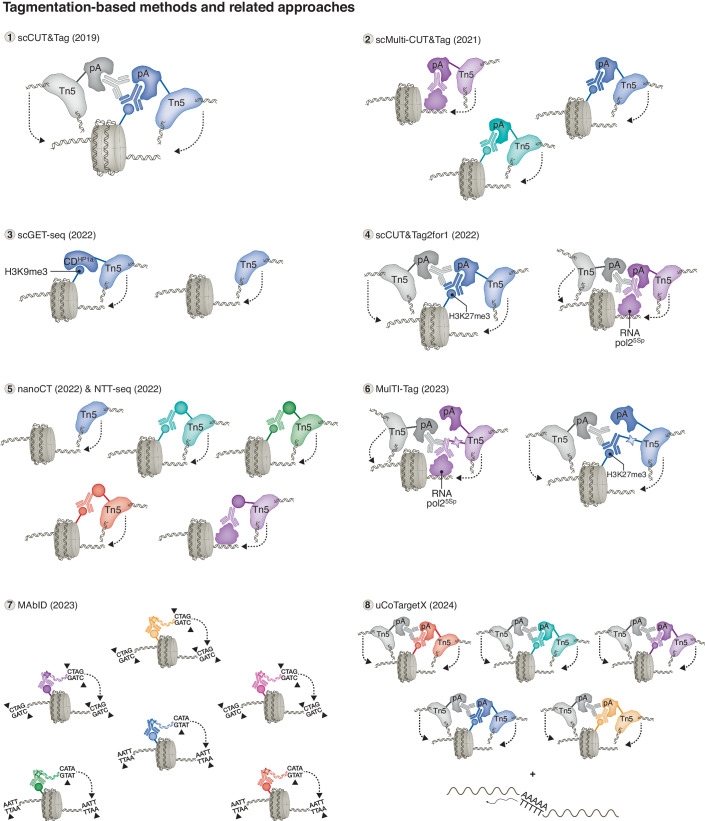
Tagmentation-based methods and related approaches. Overview of tagmentation-based approaches and related methods to study co-occurrence of chromatin features. For further details on the methods, see main text. (1) scCUT&Tag (Kaya-Okur et al, 2019). An antibody specific for a target of interest plus a secondary antibody tether and enrich the genomic region bound with pA-Tn5 transposomes that insert sequencing adapters upon activation. (2) scMulti-CUT&Tag (Gopalan et al, 2021). Antibodies coupled with pA-Tn5 complexes preloaded with sequencing adapters containing antibody-specific barcodes are tethered to the targeted epitopes. Antibody-specific barcoded adapters are inserted at the targeted genomic sites. (3) scGET-seq (Tedesco et al, 2022). Tn5 transposase and a protein fusion (TnH) composed of Tn5 with the chromodomain (CD) of HP1α are both preloaded with specific barcoded adapters. Both Tn5 and TnH are applied to cells in order to simultaneously profile open and closed chromatin. (4) scCUT&Tag2for1 (Janssens et al, 2022). Antibodies specific for RNA polymerase II with phosphoserine-5 (RNApol2S5p) and for H3K27me3 are applied to samples to tether pA-Tn5 to antibody-bound genomic regions. The tagmentation densities and fragment sizes are used to assign reads to either target. (5) nano-CT and NTT-seq (Bartosovic & Castelo-Branco, 2022; Stuart et al, 2022). Fusion proteins composed of Tn5 and a single-chain secondary nanobody that specifically recognize primary antibodies raised in different species are used to tether Tn5 to primary antibodies bound to targets of interest and insert antibody-specific barcoded adapters. (6) MulTI-Tag (Meers et al, 2023). Antibody-specific barcoded adapters are covalently conjugated to antibodies and loaded to pA-Tn5 directing the transposome to integrate barcodes at genomic binding sites of targeted proteins. (7) MAbID (Lochs et al, 2023). Antibodies specific to chromatin proteins of interest are covalently conjugated to barcoded adapters bearing motifs recognized by different sets of restriction enzymes to cleave both adapters and nearby genomic sites followed by ligation to integrate antibody-specific barcodes. (8) uCoTargetX (Xiong et al, 2024). Primary antibodies specific for different chromatin targets are coupled with pA-Tn5 that are preloaded with antibody-specific barcodes and are used to introduce barcodes and forward sequencing adapters. Secondary antibodies coupled with pA-Tn5 preloaded with reverse sequencing adapters are applied to further tagment genomic regions.

Sequential ChIP has led to the important discovery that activating and repressing histone PTMs, namely histone H3 lysine 4 and lysine 27 tri-methylation (H3K4me3 and H3K27me3, respectively), display nucleosomal co-occurrence at transcription start sites (TSSs) of key developmental transcription factor genes in mouse embryonic stem cells (mESCs) and primary human CD4+ memory T cells (Bernstein et al, 2006; Kinkley et al, 2016). These large H3K27me3-enriched regions harboring smaller H3K4me3 peaks were termed bivalent domains (Azuara et al, 2006; Voigt et al, 2012; Harikumar and Meshorer, 2015; Blanco et al, 2020; Kumar et al, 2021). Importantly, it was shown that ~15% of TSSs showing co-occurring H3K4me3 and H3K27me3 as detected by in silico dataset comparison were not detected by reChIP-seq and are thus regions of potential spatial co-occurrence rather than cellular co-occurrence (Kinkley et al, 2016). The presence of such bivalent domains was further confirmed using single-molecule combinatorial profiling of modified mononucleosomes (Fig. 2A-3) (Shema et al, 2016). This method elegantly makes use of fluorescently labeled antibodies, total internal reflection (TIRF) microscopy, and sequencing reactions to record the modification states, position, and sequence of mononucleosomes (Shema et al, 2016). In brief, the chromatin is first digested with micrococcal nuclease (MNase). Mononucleosomes are then ligated with fluorescent and biotinylated adapters, isolated, and captured on slides. Next, TIRF microscopy is used to record the nucleosome positions on the slide, as well as binding and dissociation events upon incubation with fluorescently labeled antibodies specific to histone PTMs. Finally, to determine the genomic positions of the imaged modified nucleosomes, primers are hybridized onto the biotinylated adapters and sequencing-by-synthesis is performed at each position on the slide. Applying this method to isolated histone proteins from ESCs allowed to determine the proportion of bivalent nucleosomes that bear the two modifications of interest on the same histone (Shema et al, 2016). As such, a unique feature of this approach is that it can be used to detect not only nucleosomal, but also histone co-occurrence of PTMs. However, the workflow is complex and requires specialized equipment. Further optimizations will be needed to profile low-input samples or single cells in high-throughput approaches.

Finally, a novel method based on ChIP involves a highly scalable and multiplexed platform that enables the genome-wide mapping of hundreds of proteins simultaneously (Fig. 2A-4) (preprint: Perez et al, 2023). Named chromatin immunoprecipitation done in parallel (ChIP-DIP), this technique relies on a bead labeling workflow whereby streptavidin beads are conjugated with (1) antibody-assigned barcoded and biotinylated adapters and (2) biotinylated protein G beads. In a next step, antibodies are coupled to the corresponding barcoded streptavidin–protein G bead complex by binding to protein G. A bead complex pool is generated with various antibodies and their corresponding barcoded adapters, and incubated together with fixed cells for immunoprecipitation in a single tube. Subsequently, multiple rounds of split-and-pool tagging are performed to generate a unique combinatorial index for each streptavidin bead (Quinodoz et al, 2018, 2021, 2022; preprint: Perez et al, 2023). This split-pool barcode is shared between genomic DNA and antibody-specific oligonucleotide bound to the same bead, and serves to assign the epitope barcode to its associated genomic regions. With this technique, the authors were able to generate what they qualify as “consortium level” datasets, by multiplexing over 225 antibodies specific for 160 distinct proteins in a single experiment, with target proteins encompassing histone PTMs, chromatin regulators (readers, writers, and erasers), transcription factors, and RNA polymerases (I, II including different PTM forms, and III) (preprint: Perez et al, 2023). This highly multiplexed approach shows the potential to identify regulatory features based on the combinatorial occurrence of histone PTMs. As ChIP-DIP is performed in bulk, and mapping the targets involves the matching of combinatorial barcodes from a single antibody and a genomic DNA sequence, it allows to interrogate spatial co-occurrence only.

## Section 2: Targeted DNA adenine methylation-based methods

An alternative suite of methods to map DNA-protein interactions is based on the DNA adenine methyltransferase (Dam), an *E. coli* enzyme that endogenously methylates adenine in a GATC context (Steensel and Henikoff 2000; Greil et al, 2006). Because adenine methylation (m6A) in DNA is absent in most eukaryotic cells, Dam can be used to mark regions in the genome with m6A and probe the genomic occupancy of proteins in vivo. This approach is referred to as Dam identification (DamID, Fig. 2B-1) (Steensel and Henikoff 2000; Greil et al, 2006). For this purpose, Dam is fused with a protein of interest (POI), and expressed in cells, resulting in adenine methylation on the DNA surrounding the binding sites of the POI. To determine the sites of methylation, the genomic DNA is digested by either restriction enzymes DpnI or DpnII, which specifically cleave methylated or unmethylated adenine sites, respectively, at the 5’ end of a GATC motif. The digested genomic DNA is then subjected to adapter ligation followed by adapter-mediated PCR (LMP), where DNA fragments can be amplified by PCR proportionally to the methylation frequency (Dai et al, 2000). The resulting libraries can either be used for targeted interrogation of cooperative binding at specific genomic regions with PCR or with NGS for genome-wide mapping.

DamID was later on adapted for single-cell profiling (scDamID), by sorting and distributing single cells on a 96-well plate for cell lysis and adding well-specific indexes for single-cell DamID multiplexing (Kind et al, 2015). To assess how protein-genome interactions influence gene expression in the same cell, a DamID-based method was introduced for the joint profiling of protein occupancy and messenger RNA (mRNA) expression, termed single-cell DamID with messenger RNA sequencing (scDam&T-seq, Fig. 2B-2) (Rooijers et al, 2019). Similarly to the scDamID approach, single cells sorted with fluorescence-activated cell sorting (FACS) are distributed to 384-well plates for greater throughput. To capture RNA in the same cell, polyadenylated mRNA molecules are subjected to reverse transcription, and both genomic DNA and resulting complementary DNA (cDNA) products are amplified by in vitro transcription (Rooijers et al, 2019). scDam&T was, for example, successfully applied to simultaneously profile in single cells the transcriptome and the occupancy of the PRC1 subunit RING1B. Correlation analysis of these two modalities revealed allelic bias reflecting X chromosome inactivation (Rooijers et al, 2019).

To leverage DamID and study combinatorial protein binding to chromatin, Split DamID (SpDamID, Fig. 2B-3) was developed as a protein complementation version of DamID (Hass et al, 2015). In brief, complementary halves of Dam are fused to two potentially interacting or juxtapositioned DNA-binding or chromatin proteins. If both proteins are in close proximity, a functional Dam will be reconstituted, resulting in the methylation of nearby adenines within GATC sequences. SpDamID was successfully used on as little as 100 cells but has yet to be adapted for single-cell interrogation. SpDamID has, for example, allowed to identify genomic sites of allelic co-occurrence of Notch1 and its binding partners, as well as the co-occurrence of non-interacting proteins (for example, Notch1 and Runx1 both binding to the same enhancer) (Hass et al, 2015).

A general limitation of the described DamID-based methods is that they are not directly suitable to map modified histones. To address this, EpiDamID extends the suite of DamID-based protocols (Fig. 2B-4), allowing to profile histone PTMs along with the transcriptome, in low-input samples and in single cells (Gopalan and Fazzio, 2022b; Rang et al, 2022). For this, Dam was fused to chromatin-binding molecules that can detect various histone PTMs. Three different Dam-fusion types were generated with distinct targeting domains, including (1) full-length nonhistone proteins, (2) histone modification binding domains, or (3) single-chain variable fragments, a recombinant antibody construct comprised of the variable regions of the heavy and light immunoglobulin chains connected via a peptide linker (Bird et al, 1988; Ahmad et al, 2012; Xenaki et al, 2017; Rang et al, 2022). This approach was applied for various histone PTMs, encompassing active, heterochromatin, and Polycomb marks. While the authors state that EpiDamID can be applied to any existing DamID protocol, it has yet to be adapted to study co-occurring histone PTMs (Hass et al, 2015; Rang et al, 2022). Recently, an alternative method based on the deposition of methylation marks on adenines was developed, named directed methylation with long-read sequencing (DiMeLo-seq) (Altemose et al, 2022). DiMeLo-seq makes use of a protein A (pA)–Hia5 fusion (pA-Hia5) and tethers pA-Hia5 to a POI or histone PTM via specific antibodies. Hia5 is a nonspecific deoxyadenosine methyltransferase, thus marking binding sites of the targeted POI. This approach has also yet to be adapted to study the co-occurrence of histone PTMs, DNA-binding proteins, or chromatin proteins.

## Section 3: Targeted DNA cleavage-based approaches

An early approach to direct a nuclease to cleave DNA-binding sites of a POI was named Protein Position Identification with Nuclease Tail (PIN*POINT, Fig. 2C-1) (Lee et al, 1998). With PIN*POINT, a fusion protein comprised of a non-sequence-specific nuclease domain and a POI is expressed in vivo. The nuclease is recruited by the fusion partner to chromatin and digests the DNA specifically near the POI binding sites (Dai et al, 2000). Subsequently, two methods making use of MNase were simultaneously introduced using different tethering approaches to direct DNA cleavage, namely chromatin immunocleavage (ChIC) and chromatin endogenous cleavage (ChEC) (Schmid et al, 2004).

With ChEC (Fig. 2C-2), a POI is fused with MNase and (over)expressed, directing the MNase-induced DNA cleavage near the protein binding sites. ChEC was developed for both fixed cells (termed in vitro ChEC) and for permeabilized cells in native conditions (termed in vivo ChEC). ChIC (Fig. 2C-3) is based on the fusion of immunoglobulin binding domains from the staphylococcal protein A (pA) to MNase, which is used to tether the nuclease to antibodies (Schmid et al, 2004). Specifically, fixed cells were first treated with primary antibodies specific for an epitope of interest, optionally followed by secondary antibodies, and ultimately with the pA-MNase fusion protein (with pA recognizing the primary or secondary antibody) to induce double-strand DNA cleavage in the vicinity of the target protein binding sites. Later, these methods were combined with NGS to allow for genome-wide mapping of protein binding sites, giving rise to the CUT&RUN technique (for Cleavage under targets and release under nuclease, Fig. 2C-3) and ChEC-seq, respectively (Zentner et al, 2015; Skene and Henikoff, 2017).

CUT&RUN rapidly became adopted by the research community as an efficient alternative to ChIP-based methods given its improved applicability for low-input samples (Skene and Henikoff, 2017). CUT&RUN is similar to ChIC (Fig. 2C-3): it involves immobilization of nuclei on magnetic beads and antibody-directed pA-MNase target cleavage. Chromatin fragments bound to the antibody target are cut and diffuse out of the nucleus. The released DNA can be extracted for subsequent library preparation and sequencing. It was later on further adapted to profile as little as 10 cells per experiment with ultra-low-input CUT&RUN (uliCUT&RUN, Fig. 2C-3) (Hainer et al, 2019). To achieve single-cell profiling with single-cell ChIC-seq and sort-assisted single-cell ChIC (scChIC-seq and sortChIC, respectively, Fig. 2C-4), single cells are sorted onto 384-well plates by FACS to perform the DNA cleavage reaction in individual wells, followed by fragment indexing with cell-specific barcodes, before pooling for amplification of the libraries (Ku et al, 2019; Zeller et al, 2023). Furthermore, the addition of an in situ reverse transcription step has provided the possibility to capture mRNAs from their poly-A tail in the same cell to jointly measure gene expression, named single-cell profiling of chromatin occupancy and RNA sequencing (scPCOR-seq, Fig. 2C-5) (Pan et al, 2022).

To allow for the multiplexing of target histone PTMs based on the ChIC principle, a strategy has been developed that involves an experimental and bioinformatics framework named single-cell chromatin immunocleavage and unmixing sequencing (scChIX-seq, Fig. 2C-6) (Yeung et al, 2023). In scChIX-seq, three sortChIC maps are generated: two maps from two distinct histone PTMs resulting from incubating each antibody separately, and one map resulting from double-antibody incubation targeting both histone PTMs simultaneous (Yeung et al, 2023; Zeller et al, 2023). The individual histone PTM profiles are used as training sets to deconvolve the multiplexed profile dataset. Consequently, the modeling allows to infer histone PTM profiles in single cells for both mutually exclusive sites of enrichment as well as sites of co-occurrence (Yeung et al, 2023). In addition, because the scChIX-seq workflow includes a FACS step, it is possible to purify samples based on cell types (Ku et al, 2019; Yeung et al, 2023; Zeller et al, 2023). By studying different pairs of either mutually exclusive or co-occurring PTMs, the scChIX-seq framework allowed to define cell-specific histone PTM landscapes (H3K27me3–H3K9me3), that distinguish cell types with more confidence than based on mRNA abundances alone (H3K4me1–H3K27me3), quantify overlapping modifications (H3K36me3–H3K9me3) across cell types during organogenesis, and apply pseudotime analysis to infer chromatin velocity throughout an in vitro macrophage differentiation timecourse (H3K36me3–H3K4me1) (Yeung et al, 2023). While scChIX-seq makes it possible to deconvolve multiplexed histone PTM profiles, it has a trade-off of a limited spatial resolution and thus on the applicability to study cellular co-occurrence.

## Section 4: Targeted tagmentation-based methods and related approaches

During the past few years, methods making use of the Tn5 transposase for epigenomic applications have rapidly evolved, particularly for single-cell profiling. These toolkits are largely based on chromatin under targets and tagmentation (CUT&Tag, Fig. 3-1), antibody-guided chromatin tagmentation (ACT-seq), and combinatorial barcoding and targeted chromatin release (CoBATCH) (Carter et al, 2019; Kaya-Okur et al, 2019; Wang et al, 2019). CUT&Tag was initially developed for low-input and single-cell approaches, and showed utility for profiling histone PTMs, RNA Polymerase II, and transcription factors (Kaya-Okur et al, 2019). The basic principle involves the fusion of protein A with an hyperactive Tn5 transposase (pA-Tn5) that is loaded with sequencing adapters, termed transposome, and tethered with antibodies (that bind to pA) against a target of interest (Reznikoff, 2003; Picelli et al, 2014). The Tn5-catalyzed targeted tagmentation reaction is activated with the addition of magnesium ions which causes the integration of adapters into the DNA at antibody-bound regions, generating sequencing-ready libraries within a day of wet lab work (Kaya-Okur et al, 2019). The protocol was originally implemented to profile from 100,000 cells and down to 60 cells in bulk. Using a nano-dispensing system to distribute cells on a 5184-nanowell chip, and indexed primers, epigenomic profiling with CUT&Tag was also achieved at single-cell resolution (Kaya-Okur et al, 2019). With variations in the tagmentation reaction conditions, the transposome can be redirected to generate chromatin accessibility profiles in a single tube and even on a home workbench, referred to as Cleavage Under Targeted Accessible Chromatin (CUTAC) (Henikoff et al, 2020, 2021). Single-cell CUT&Tag (scCUT&Tag) was later on adapted in combination with the droplet-based 10x Genomics platform with optimization for complex tissues (Bartosovic et al, 2021). In addition, scCUT&Tag was recently combined with cell surface protein abundance measurement (scCUT&Tag-pro) (Zhang et al, 2022). In the same article, a computational framework termed single-cell ChromHMM (scChromHMM) was also introduced, allowing to integrate scCUT&Tag-pro datasets of six distinct histone PTMs, and predict chromatin state annotations at single-cell resolution (Zhang et al, 2022). This approach further highlights the added benefit of integrating multiple datasets for chromatin state inference, but still lacks direct detection of chromatin feature co-occurrence.

The first method that was described to directly profile multiple targets based on the CUT&Tag procedure was multi-CUT&Tag (Fig. 3-2) (Gopalan et al, 2021; Gopalan and Fazzio, 2022a). This method uses a histidine-tagged version of the pA-Tn5 fusion that allows to purify the transposome complexes pre-coupled with antibodies specific for the epitopes of interest. To incorporate antibody-specific barcodes during tagmentation, the pA-Tn5 fusion proteins pre-coupled with antibodies are also preloaded with antibody-specific barcodes flanked by transposase-compatible adapters and sequences suitable for NGS for the production of sequencing-ready libraries. Sequencing reads can then be demultiplexed based on the antibody-assigned barcodes to determine binding profiles of each target, while simultaneously defining sites of nucleosomal co-occurrence. This approach has also been adapted for examination in single cells with the 10x Genomics platform (scMulti-CUT&Tag) (Gopalan et al, 2021; Gopalan and Fazzio, 2022a). Unbiased clustering performed with the simultaneously profiled H3K27me3 and H3K27ac marks enhanced the identification of distinct cell types from a mixture of ESCs and trophoblast stem cells (TSCs) (Gopalan et al, 2021).

To jointly probe open and closed chromatin states in the same cell, single-cell genome and epigenome by transposases sequencing (scGET-seq, Fig. 3-3) was developed (Tedesco et al, 2022). This tagmentation-based method makes use of a hybrid transposase (TnH) where Tn5 is fused with the chromodomain (CD) of the heterochromatin protein-1alpha (HP1α), a reader for H3K9me3. By sequentially treating permeabilized cells with Tn5 and TnH loaded with two different sets of barcoded indexes, the authors were able to probe accessible and compacted chromatin (Tedesco et al, 2022). This approach was adapted to achieve single-cell resolution by combining with the 10x Genomics droplet-based protocol, and has the ability to also call copy number variant (CNV) events as well as single-nucleotide variants (SNVs). Another alternative to jointly profile open and closed chromatin is single-cell CUT&Tag2for1 (scCUT&Tag2for1, Fig. 3-4), which extends from CUTAC (Henikoff et al, 2020, 2021; Janssens et al, 2022). Here, the Tn5 transposase is directed to antibodies specific for RNA polymerase II with phosphoserine-5 (Pol2S5p) and a “repressive” histone PTM (H3K27me3) with mutually exclusive binding profiles. Sequencing reads are then deconvoluted computationally to generate single-cell maps of both active and repressed chromatin, by accounting for tagmentation densities and fragment length differences. Single-cell profiles are generated with the use of a microfluidic chip to add cell-specific barcodes and amplify libraries. Both scGET-seq and scCUT&Tag2for1 can serve to simultaneously profile open and closed chromatin states and are currently not directly applicable to study the co-occurrence of chromatin features (Janssens et al, 2022; Tedesco et al, 2022).

Two novel and highly similar approaches now employ nanobody-tethered transposases to profile up to three modalities in bulk or single cells with starting material ranging from 25 K to 1 M cells, namely the nanobody-CUT&Tag (nano-CT, Fig. 3-5) and the nanobody-tethered transposition followed by sequencing (NTT-seq, Fig. 3-5) (Bartosovic and Castelo-Branco, 2022; Stuart et al, 2022). These methods make use of several sets of recombinant fusion proteins that are composed of the Tn5 transposase and nanobodies (nano-Tn5). Nanobodies are small single polypeptide chain antibodies that can strongly and stably bind to their target over a range of temperature and pH conditions. The engineered nano-Tn5 are made specific for a set of immunoglobulins from different species or for different IgG subtypes directed against the epitope of interest. Similarly to scMulti-CUT&Tag and scGET-seq, the transposases are loaded with different sets of barcoded adapters for the corresponding antibody/species and therefore insertion events originating from the different nanobody-Tn5-antibody complexes can be demultiplexed based on these barcoded sequences. Assaying combinations of chromatin features enabled improved identification of cell types (Stuart et al, 2022). In addition, chromatin remodeling events were captured across the differentiation progression of human bone marrow cells (Stuart et al, 2022) and mouse brain oligodendrocyte cells (Bartosovic and Castelo-Branco, 2022) through profiling of H3K27ac and H3K27me3. The multimodal feature of these approaches allows to identify the trajectory of differentiating cells with their corresponding chromatin states, as well as additional chromatin state dynamics. Specifically, combining multimodal chromatin profiling by nano-CT and chromatin velocity modeling from differentiating cells in the mouse brain (also including ATAC signal along with H3K27ac and H3K27me3) was used to infer differentiation kinetics, and to identify genes that regulate the chromatin landscape (Bartosovic and Castelo-Branco, 2022).

To circumvent potential cross-contamination of antibody-specific adapters, an alternative approach made use of the principle of chromatin integration labeling (ChIL), where pA-Tn5 is coupled with antibodies that are covalently conjugated with antibody-specific adapters (termed ChIL probe). This approach combined immunostaining, transposase tagging, followed by linear amplification, for profiling up to three targets simultaneously (Schmidl et al, 2015; Harada et al, 2019; Handa et al, 2020; Meers et al, 2023). With Multiple Target Identification by Tagmentation (MulTI-Tag, Fig. 3-6), permeabilized cells are immunostained and tagmented with different ChIL probes for each target sequentially, and a final tagmentation step is carried out with secondary antibodies conjugated with sequences complementary to reverse primers to increase fragments per cell (Meers et al, 2023). To achieve single-cell profiling, samples are first processed in bulk as described above, and the final tagmentation step is performed on a 96-well plate with each well containing transposomes with a unique barcode. Finally, nuclei are pooled back together and then distributed on a microfluidic chip for combinatorial indexing and amplification, generating cell-specific barcode combinations. Analysis of MulTI-Tag maps in H1 human ESCs at different differentiation stages revealed that the integration of data from the multifactorial chromatin states improved developmental trajectories inference (Meers et al, 2023). The authors also investigated the allelic and nucleosomal co-occurrence of different targets, and intriguingly detected sites co-enriched with H3K27me3-H3K36me3 on different nucleosomes but within the same gene (Meers et al, 2023).

Recent advances in single-cell multi-omics technologies have pushed the limits for profiling multiple targets, with the possibility to jointly map up to six epitopes in a single experiment. First, for Multiplexing Antibodies by barcode Identification (MAbID, Fig. 3-7), nuclei are isolated and lightly fixed, treated with primary antibodies with different host origin, incubated with secondary antibodies that are covalently conjugated with barcoded adapters and specific for each primary antibody, and sorted with FACS (Lochs et al, 2023). In lieu of exploiting the Tn5 transposase to tagment the targeted genomic regions, the genome as well as the antibody-assigned adapters are digested with restriction enzymes, generating overhang templates that are compatible for subsequent ligation. Including additional sets of restriction enzymes allowed to increase the combinatorial space for antibody multiplexing. To further increase the number of multiplexed targets in a single experiment and overcome the dependency on antibodies of varied host origins, the authors applied the antibody–adapter conjugation directly to the primary antibodies (Lochs et al, 2023). While this lowered the signal-to-noise ratio, the multiplexing capacity is greatly enhanced, and the simultaneous mapping of six epitopes encompassing all major chromatin types was validated. MAbID was also adapted for the investigation of single cells (scMAbID) by integrating the use of 384-well plates and liquid-handling robotics, and of primary tissues. Similar to the other multimodal methods described here, scMAbID was shown to improve the identification of different cell types with multiplexed measurements in comparison with single profiles. scMAbID was also applied to study allele-specific chromatin dynamics associated with X chromosome inactivation during development (Lochs et al, 2023).

Finally, recent technological advancements allow for the profiling of up to five histone PTMs at single-cell level by two related methods: (ultra)high-throughput Combined TAgmenting enRichment for multiple epiGEneTic proteins in the same cell (uCoTarget), and uCoTargetX, which allows for the simultaneous profiling of the transcriptome in the same cell (Fig. 3-8) (Xiong et al, 2024). This strategy involves the sequential treatment of lightly fixed and permeabilized cells first with pA-Tn5 complexes coupled to different antibodies and preloaded with antibody-assigned barcodes containing a first set of indexed reverse adapter sequences. Cells are next treated with a second set of transposomes coupled to secondary antibodies and preloaded with the second set of indexed forward adapter sequences to facilitate further tagmentation at genomic regions enriched for targeted chromatin proteins and therefore increase the number of fragments generated per cell. For single-cell assays, the authors introduced a split-pool combinatorial indexing strategy into the protocol to generate cell-specific barcoding that vastly expands the scalability of the assay (Rosenberg et al, 2018; Ma et al, 2020). Integrating both epigenomic and transcriptomic modalities improved accuracy for cell type identification.

## Section 5: Selecting the method of choice: some pros and cons

The following section compares and contrasts approaches that can simultaneously profile multiple chromatin features genome-wide. We discuss considerations regarding the applicability of the methods, the ease of their implementation, the interpretation of the generated data, and possible additional features. Table EV1 contains a summary of the characteristic features of the relevant methods, along with selected advantages and limitations.

### Application

Based on the research question, the methods described here can serve to either interrogate (1) solely sites of co-occurrence, such as with sequential ChIP-based approaches or with SpDamID (Hass et al, 2015; Kinkley et al, 2016; Weiner et al, 2016; Seneviratne et al, 2024), or alternatively (2) obtain the genome-wide occurrence of multiple chromatin features in a given experiment, including the localization of individual features and their colocalization (Gopalan et al, 2021). Most of the mapping approaches described here can **map** multiple chromatin features in parallel in one experiment, whereas a subset of the methods is based on **inferring** co-occurrence, either by using available datasets of singly assayed targets (in silico inference), or via unmixing (deconvolution) of reads such as with scChIX-seq (see Table EV1: Deconvolution of target profile) (Yeung et al, 2023). Indeed, in silico inference can be useful to identify candidate proteins or genomic sites, but subsequent validation of cellular co-occurrence is required (Fig. 1B). Likewise, deconvolution of reads originating from multiplexed targets may be more challenging when the assayed targets are not mutually exclusive or show highly similar enrichment profiles on the genome.

The approaches vary in terms of the **possible targets** studied (see Table EV1: Types of targets assayed). Some methods were, for example, designed to specifically target simultaneously open and closed chromatin such as with scGET-seq and scCUT&Tag2for1 (Janssens et al, 2022; Tedesco et al, 2022). Other approaches may be more suitable to study histone PTMs, such as with single-molecule combinatorial nucleosome profiling and scChIX-seq (Shema et al, 2016; Yeung et al, 2023), whereas certain methods can only profile nonhistone proteins as with SpDamID (Hass et al, 2015). Most ChIP-based and tagmentation-based methods are suitable for assaying both histone PTMs and nonhistone proteins simultaneously.

The **multiplexing capacity** to assay multiple chromatin features in a single experiment depends on the underlying principle of the approach used (see Table EV1: Number of targets assayed). For example, sequential ChIP-based approaches may not be suitable for more than two targeted chromatin features as increasing the number of immunoprecipitation steps will further reduce the yield and these approaches already require relatively high amounts of starting material (Kinkley et al, 2016; Weiner et al, 2016; Seneviratne et al, 2024). Some methods were specifically designed to assay two targets only, such as those for open and closed chromatin (scGET-seq and scCUT&Tag2for1) (Janssens et al, 2022; Tedesco et al, 2022), or SpDamID that can only profile two nonhistone proteins (Hass et al, 2015). With scChIX-seq, three sortChIC maps are already required to assay two histone PTMs simultaneously (one map from two multiplexed targets, and two separate maps of individual targets for deconvolution) making it experimentally and computationally complex to increase the number of targets assayed (Yeung et al, 2023). Nanobody-tethered tagmentation approaches (nano-CT and NTT-seq) are currently limited to two (plus open chromatin profiling with nano-CT) or three targets assayed in a single experiment, respectively, because the antibodies used need to be raised in different host species (Bartosovic and Castelo-Branco, 2022; Stuart et al, 2022). In general, methods that insert target-specific barcodes are applicable to an increased number of assayed targets, such as multi-CUT&Tag and MulTI-Tag (Gopalan et al, 2021; Gopalan and Fazzio, 2022a; Meers et al, 2023). Currently, the methods that have proven the greatest multiplexing capacity are uCoTarget with 5 targets, MAbID with 6 targets, and ChIP-DIP with hundreds of targets (>225) (Lochs et al, 2023; preprint: Perez et al, 2023; Xiong et al, 2024).

Based on the research question one needs to also consider the **input sample** requirements and the **data output** generated. Sample conditions range from native, to light or strong fixation, which can be tailored based on the sample origin and the targets assayed (for more details, see Table EV1: Sample conditions). Although some methods have only been tested in cultured cells, optimized sample preparation and tissue dissociation procedures, along with optimized fixation conditions, should make many methods applicable to tissues or clinical samples (see Table EV1: Sample type tested). Of note, methods that require expression of a fusion protein such as with SpDamID are generally not suitable for processing clinical samples or most non-cultured sample types (Hass et al, 2015).

Recent advances with ChIP-reChIP have allowed to reduce the number of cells required as starting material (from tens of million to two million cells) (Seneviratne et al, 2024), but most ChIP-based methods still require a higher number of cells than enzyme-tethering approaches (see Table EV1: Input; Cells loaded; Cells recovered). Indeed, most of the enzyme-tethering approaches presented here have been adapted for **single-cell** or **ultra-low-input** profiling. Several optimizations particularly with tagmentation-based methods have improved the tagmentation efficiency and thus the number of fragments per cell (see Table EV1: Fragments per cell; Fragments per cell per target), for example by performing the tagmentation reactions sequentially (rather than simultaneously), beginning with the least abundant target (see Table EV1: Multi-targeting approach) (Stuart et al, 2022; Meers et al, 2023; Xiong et al, 2024). Additionally, using a second set of transposomes tethered by a secondary antibody increases the number of enzyme complexes recruited thereby facilitating further tagmentation (see Table EV1: Antibody targeting) (Meers et al, 2023; Xiong et al, 2024). Another elegant strategy is exploited in the nano-CT protocol: it involves linear amplification for library construction, which obviates the need for two nearby and properly oriented tagmentation events for fragment amplification (Bartosovic and Castelo-Branco, 2022). While this strategy results in a greater number of fragments per cell allowing for a lower amount of starting material to be used, a trade-off can be a slightly higher tagmentation background and therefore a lower fraction of reads in peaks (FRiP) (Bartosovic and Castelo-Branco, 2022).

### Accessibility

The implementation of these toolkits in a laboratory may come with high costs as well as certain challenges related to specialized equipment, compatibility with commonly used platforms, intricate experimental steps, or complex analysis workflows. In particular, different methods for single-cell profiling often involve the use of costly and specialized platforms (see Table EV1: Single-cell approach). These specific single-cell profiling approaches as well as more accessible and scalable alternatives are described in Box 1.

It is important to note that a subset of methods is currently not compatible with standard illumina protocols and require custom sequencing primers, which often prevents a pooling of samples from different experiments (see Table EV1: Compatibility with illumina standard protocols). These include multi-CUT&Tag, scGET-seq, nano-CT and NTT-seq (Gopalan et al, 2021; Bartosovic and Castelo-Branco, 2022; Gopalan and Fazzio, 2022a; Stuart et al, 2022; Tedesco et al, 2022). However, as performed with uCoTarget, adaptations of adapter sequences used are possible in order to obtain libraries compatible with standard Iillumina protocols (Xiong et al, 2024).

Several workflows require **additional reagent preparation** steps or **specialized equipment** (see Table EV1: Extra reagent preparation and specialized equipment required). An example is the multi-CUT&Tag protocol, which makes use of a specific histidine-tagged pA-Tn5 fusion that has to be expressed and purified (Gopalan et al, 2021; Gopalan and Fazzio, 2022a). Along the same lines, ChIP-DIP requires the preparation of labeled beads for each antibody used (preprint: Perez et al, 2023). Similarly, MulTI-Tag and MAbID make use of a ChIL probe (Harada et al, 2019; Handa et al, 2020) that requires covalent conjugation of antibodies to barcoded adapters (Lochs et al, 2023; Meers et al, 2023). This step was introduced in order to reduce off-target signals, however Xiong et al, have recently suggested that these antibody conjugates may actually hinder epitope recognition, particularly with histone PTMs (Xiong et al, 2024). Methods that involve the expression of a fusion protein such as with SpDamID require testing and optimization for each fusion protein as well as for each new experimental system (Gopalan and Fazzio, 2022b; Hass et al, 2015). Lastly, several methods require additional specialized equipment, such as TIRF microscopy (Shema et al, 2016), FACS devices, or microfluidics instruments (Gopalan et al, 2021; Bartosovic and Castelo-Branco, 2022; Gopalan and Fazzio, 2022a; Janssens et al, 2022; Stuart et al, 2022; Tedesco et al, 2022; Lochs et al, 2023; Meers et al, 2023; Yeung et al, 2023).

Box 1 Approaches to achieve single-cell -omics profiling and alternatives with microfluidics-free methods Since the establishment of the first approach for cell-specific barcoding (Islam et al, 2011), the field of single-cell -omics profiling has rapidly evolved and been adapted for various modalities (Vandereyken et al, 2023). The novel methods used employ different strategies (plate-based, droplet-based, or combinatorial indexing) to barcode individual cells for subsequent pooling and amplification of the libraries ready for high-throughput next-generation sequencing.

Early approaches to achieve single-cell barcoding involved picking individual cells, distributing them on a 96-well PCR plate preloaded with buffers for processing and barcoding (Islam et al, 2011). Nowadays, **plate-based** methods (A) take advantage of automated strategies such as FACS or multi-sample nano-dispensing platforms to distribute individual cells into wells, where the experimental reactions take place, greatly reducing labor and improving throughput (Fan et al, 2015; Goldstein et al, 2017; Mereu et al, 2020). In addition, a FACS step can enrich specific cells, reducing sequencing costs. Similarly, a built-in imaging software with nano-dispensing instruments also allows to visualize wells of the microchip, and thus providing control over the selection of wells to use for downstream processing. Of note, these approaches have limited scalability and require robotics for high-throughput liquid-handling and processing of samples (Mereu et al, 2020). Nevertheless, they have been adopted in several workflows presented in this review (Janssens et al, 2022; Lochs et al, 2023; Meers et al, 2023; Yeung et al, 2023).

**Droplet-based** single-cell profiling methods (B) make use of a microfluidics system that enables the formation of droplets through water-in-oil emulsion (Salomon et al, 2019). These droplets serve as a reaction chamber where individual cells or nuclei are encapsulated with barcoding beads. Following barcoding, amplification, and library preparation, fragments bearing cell-specific barcodes are sequenced and bioinformatically demultiplexed for single-cell interrogation. Important advantages with this approach include the automation of the procedure, the increased throughput while simultaneously improving sensitivity and minimize reagent use (Salomon et al, 2019). This principle led to the establishment and wide adoption of technologies such as Drop-seq, inDrop, and Chromium 10X/GemCode (Klein et al, 2015; Macosko et al, 2015; Zheng et al, 2017). Single-cell profiling was initially developed for single-cell RNA sequencing (scRNA-seq) and has since then been adapted for other modalities where cells/nuclei are used as the reaction chamber for experimental processing (for example tagmentation), as with most methods presented in this review (Gopalan et al, 2021; Bartosovic and Castelo-Branco, 2022; Stuart et al, 2022; Tedesco et al, 2022). Commercial systems have streamlined the workflow thereby enabling the wide adoption of these approaches, but with limited flexibility for optimizations or adapting protocols to broaden the applicability and often high costs. More recently, a microfluidics-free droplet-based method has been established as an alternative, where droplets are generated simply with a vortexer, named particle-templated instant partition sequencing (PIP-seq) (Clark et al, 2023).

**Combinatorial indexing** (C) involves the sequential addition of barcodes to RNA or DNA molecules, generating cell-specific barcode combinations (Rosenberg et al, 2018; Cao et al, 2017). In brief, processed samples (native or fixed, cells or nuclei) are distributed into a 96-well plate where each well contains a unique barcoded adapter, to ligate the barcoded oligonucleotides to chromatin or cDNA fragments (Rosenberg et al, 2018; Ma et al, 2020). Samples are then pooled back together, and these split-and-pool steps are repeated three to four times, generating an exponential number of cell-specific barcode combinations. This approach comes with great advantages over microfluidics-based methods and plate-based methods given it obviates the need for partitioning individual cells into droplets or microwells. Combinatorial barcoding therefore offers greater flexibility in terms of scalability potential and adapter compatibility, and it does not require any specialized equipment. Split-pool combinatorial indexing was initially developed for scRNA-seq and has now been adopted to simultaneously profile multiple chromatin features along with the transcriptome in the same single cells (Zhu et al, 2019; Ma et al, 2020; Xiong et al, 2024), providing a scalable and flexible alternative over expensive kits and specialized equipment.
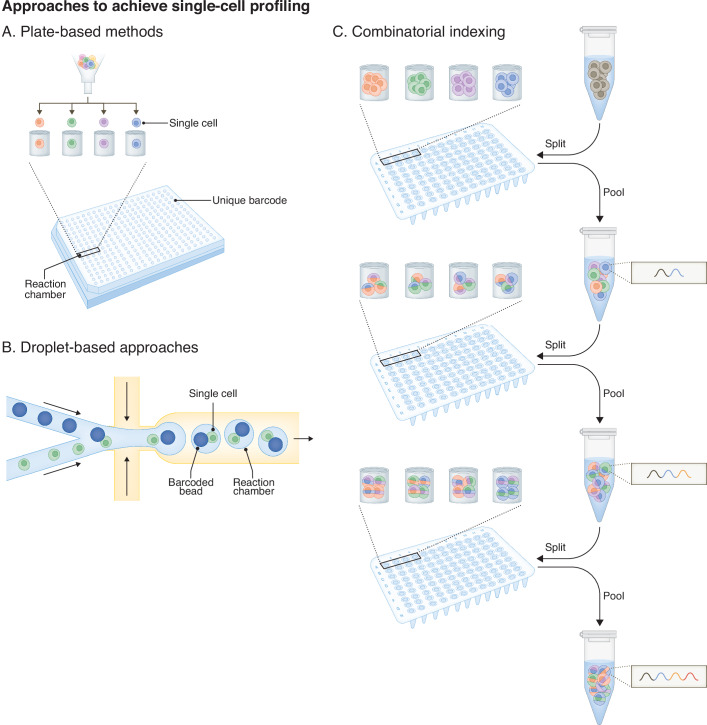

### Interpretation

Several factors need to be considered regarding the interpretation of the data generated, such as the specificity of the targeting approach, the intrinsic biases inherent to the technique used, the potential background, and the level of co-occurrence detection (see Table EV1: Data interpretation, Signal resolution, and Co-occurrence level detected).

The **specificity** of the targeting strategy depends heavily on the antibodies used as commercial antibodies vary in their specificity and often even from batch to batch. In addition, covalent labeling with a barcoded oligonucleotide (ChIL probe) (Harada et al, 2019; Handa et al, 2020; Lochs et al, 2023; Meers et al, 2023) may result in epitope masking (Xiong et al, 2024). Many antibodies were originally selected for specificity in ChIP and their specificity in other assays still has to be validated. Several approaches such as DamID-based methods or scGET-seq circumvent the use of antibodies (Hass et al, 2015; Tedesco et al, 2022). However, testing is required to ensure the tethered Dam does not interfere with the activity of the target proteins (Greil et al, 2006). In addition, Dam is known to have a **bias** toward accessible chromatin, and Dam expression for extended periods of time can result in off-target adenine methylation (Greil et al, 2006). This can be controlled for by comparing the enrichment signal with that of an unfused Dam (Greil et al, 2006). Similarly, tagmentation-based approaches are known to have inherent accessibility bias (Steiniger et al, 2006; Buenrostro et al, 2013; Kaya-Okur et al, 2019). Higher salt conditions during tagmentation steps have been performed to mitigate this bias, but it still remains unclear whether these buffer conditions are sufficient to completely overcome open chromatin biases (preprint: Meng and Yi 2021).

**Background** signal can for example arise from residual activity of untethered enzymes. ChIP-based methods are known to show higher background signals with potential for epitope masking resulting from crosslinking conditions (Park et al, 2013; Teytelman et al, 2013; Meyer and Liu, 2014; Jain et al, 2015; Baranello et al, 2016). N-ChIP can improve sensitivity, specificity and overall efficiency (Brind’Amour et al, 2015). Anti-IgG controls can be used to account for signal background in ChIP-based methods, but such negative controls perform poorly with tagmentation-based experiments (Gopalan and Fazzio, 2022a). With DamID approaches, the in vivo expression of Dam can result in spurious adenine methylation with levels that gradually accumulate in accessible chromatin, and the background level greatly differs between different Dam fusions (Greil et al, 2006; Rang et al, 2022).

The signal **resolution** and **co-occurrence** detection both depend on the fragmentation strategy, the specific motif recognition of the enzyme used, the “tagging” approach, and the binning strategy during analysis (see Table EV1: Signal resolution and co-occurrence level detected).

ChIP-based methods that make use of sonication for chromatin shearing will give rise to broader fragment sizes (and therefore lower resolution) (Kinkley et al, 2016; Weiner et al, 2016), in comparison with MNase-digested chromatin which can be easily adapted to obtain predominantly mononucleosome-sized fragments (Seneviratne et al, 2024; Shema et al, 2016). For this reason, the level of genomic co-occurrence detected can range from allelic to nucleosomal level based on the fragment size with reChIP-seq, co-ChIP-seq, and ChIP-reChIP (Kinkley et al, 2016; Weiner et al, 2016; Seneviratne et al, 2024). Because single-molecule combinatorial nucleosome profiling allows to profile mononucleosomes and individual histone proteins, its detection resolution is at the histone co-occurrence level (Shema et al, 2016). In contrast, although the fragment sizes range from 150–700 bp, ChIP-DIP can only study spatial-level co-occurrence given the tagging strategy is performed in bulk (preprint: Perez et al, 2023).

With SpDamID, as Dam specifically methylates adenine in the GATC motif, the resolution is limited to 1–2 kb, depending on the frequency distribution of GATC motifs in the genome, allowing in most cases to measure co-occurrence at the allelic level (Greil et al, 2006; Hass et al, 2015; Gopalan and Fazzio, 2022b; Rang et al, 2022). Similarly, MAbID can detect allelic co-occurrence, and its resolution is limited by the target motifs of the restriction enzymes used to insert antibody-specific barcodes and was determined to be 1–2 kb broader than with ChIP-seq (3–4 kb for H3K4me3, 7–8 kb for H3K27me3) (Lochs et al, 2023).

With scChIX-seq, even though the MNase-digested chromatin fragments predominantly reflect mononucleosomes in size, the signal can only be at locus-resolution (Yeung et al, 2023). This is because even though a single nucleosome bears multiple modifications, it will only be cut once. As this approach does not involve any “tagging” (as for example in tagmentation-introduced barcodes), the read fragments need to be merged into windows of 5–50 kb in size. Furthermore, because with scChIX-seq the profiles are deconvoluted, the genomic co-occurrence can only be inferred rather than directly measured (Yeung et al, 2023).

To allow the detection of co-occurrence on a single fragment with scMulti-CUT&Tag, Gopalan and al. have designed a technique that inserts antibody-specific barcoded adapters, where co-existing targets will result in fragments flanked by two different barcodes (mixed insertion events) whereas fragments flanked with the same barcode would arise from DNA bound to an individual assayed target (single insertion event). While the genomic resolution of the co-occurrence detected has not been specifically tested, the size distribution of the reads arising from mixed insertion events were representative of nucleosomal and sub-nucleosomal fragments (Gopalan and Fazzio, 2022a; Gopalan et al, 2021). For this reason, both scMulti-CUT&Tag, MulTI-Tag, and uCoTargetX can detect allelic or nucleosomal co-occurrence (Gopalan et al, 2021; Gopalan and Fazzio, 2022a; Meers et al, 2023; Xiong et al, 2024). Importantly, most methods are limited by two targets that can be detected per fragment (for example with methods that insert antibody-specific barcodes on each end of a fragment). Binning strategies allow to mitigate this drawback but result in a decreased resolution as a trade-off.

### Additional features

Finally, several workflows allow to assay additional features/modalities and thus broaden the method’s applicability (see Table EV1: Additional features). Along with profiling DNA-binding proteins or histone PTMs, tagmentation-based approaches have the possibility to assay transposase accessible chromatin (ATAC) in parallel, as exemplified with scGET-seq and nano-CT (Buenrostro et al, 2015; Bartosovic and Castelo-Branco, 2022; Tedesco et al, 2022). In addition, several toolkits have integrated a reverse transcription step in order to jointly capture mRNAs along with chromatin features in single cells, including scDam&T, SHARE-seq (simultaneous high-throughput ATAC and RNA expression with sequencing), Paired-tag, CoTECH (combined assay of transcriptome and enriched chromatin binding), SET-seq (same cell epigenome and transcriptome sequencing), scPCOR-seq, and more (Rooijers et al, 2019; Ma et al, 2020; Sun et al, 2021; Xiong et al, 2021; Zhu et al, 2021; Pan et al, 2022). More recently, joint gene expression measurement with multiple chromatin features has been described with uCoTargetX (Xiong et al, 2024). Such approaches have the potential to unambiguously link chromatin features with transcriptional output. Other methods that have a FACS step included in their framework such as NTT-seq, scChIX-seq, and MAbID, can simultaneously profile cell surface protein expression and thereby enrich for specific cell types (Stuart et al, 2022; Lochs et al, 2023; Yeung et al, 2023). Lastly, because Dam-induced adenine methylation deposition is stable and cumulative, the DamID-based toolkits offer the possibility to track epigenetic histories over varying time windows (Kind et al, 2013, 2015; Park et al, 2019; Rooijers et al, 2019; Gopalan and Fazzio, 2022b; Rang et al, 2022).

## Conclusions and perspectives

Over two decades ago, the “histone code” hypothesis proposed that the co-occurrence of multiple histone PTMs could give rise to unique biological outcomes (Turner, 1993, 2000; Strahl and Allis, 2000; Jenuwein and Allis, 2001). Since then, it has become evident that multiple chromatin features, not only histone PTMs, but also DNA-binding proteins, chromatin proteins, DNA modifications, or chromatin accessibility, co-occur and act cooperatively to dictate specific biological outcomes.

The number of approaches to profile various epigenomic modalities in single cells has rapidly evolved in the recent years and has revealed the ever-growing complexity of biological systems and heterogeneity of cellular states (Vandereyken et al, 2023). More recently, advances in methodological toolkits as described in this review allow now to profile hundreds of multiplexed chromatin proteins in bulk samples (preprint: Perez et al, 2023), and multiple chromatin features in single cells (Gopalan et al, 2021; Bartosovic and Castelo-Branco, 2022; Janssens et al, 2022; Stuart et al, 2022; Tedesco et al, 2022; Lochs et al, 2023; Meers et al, 2023; Yeung et al, 2023; Xiong et al, 2024).

The currently available toolkits described in this review have so far mainly been applied to improve the identification of cell types, or to define cellular states, trajectories, and chromatin velocity, by leveraging the multimodal features of the approaches. Moving forward, the potential of these toolkits in profiling varying levels of chromatin feature co-occurrence will be used to address further biological questions. In this context, major challenges in studying the combinatorial occurrence of chromatin features involve the applicability of the toolkits to study low-abundant targets and rare cell populations (see also Box 2 for more information). Specifically, optimizations will be required to overcome issues with data sparsity, limited coverage, and enhance the throughput of single-cell profiling, as well as to increase the number of chromatin targets simultaneously multiplexed per experiment. In the long run, these advances will allow to unambiguously link combinatorial chromatin feature occurrences to transcriptomic states and the systematic decoding of chromatin features in health and disease.

Box 2 In need of answers

i. How does the co-occurrence of chromatin features fine-tune gene expression?
  *Simultaneously profiling the transcriptome along with various chromatin features in the same cell will allow to unambiguously correlate chromatin states with gene expression outputs*.
ii. What is the functional relevance of the combinatorial occurrence of histone PTMs, DNA-binding proteins, and/or chromatin proteins?
  *Genetic, epigenetic, pharmacological, or environmental/metabolic manipulations will be useful to dissect the role of different combinations of chromatin features on cellular function and gene expression in the context of a wide range of biological processes such as development, stemness, differentiation, metabolism, in health and disease*.
iii. How do the distinct levels of co-occurrence (spatial, cellular, allelic, nucleosomal, and histone) contribute to the regulation of biological processes?
  *Combinations of different approaches, their future refinements, and novel techniques, will allow greater resolution, the profiling of an increasing number of targets, in lower cell numbers and thus providing new insights into the biological relevance of chromatin feature co-occurrence*.
iv. What aspects of the currently available toolkits require improvements?
  *Combining cellular co-occurrence profiling along with measurement of additional features such as of the nascent transcriptome, 3D-chromatin architecture, or temporal information, will allow to directly correlate combinatorial occurrence with different genomic outputs. The described toolkits have already shown that multimodal profiling allows to more accurately define cellular states and chromatin landscape, and that multifactorial data integration enhances the study of chromatin velocity and trajectories along developmental and differentiation progression. Increasing the number of targets or genomic features simultaneously assayed within each cell or the temporal resolution will allow to further interrogate the heterogeneity of cellular types and states and provide increasingly complex maps of chromatin features. Improving the sensitivity of the methods in order to obtain a higher number of reads per cell will also be beneficial to mitigate the data sparsity inherent to single-cell profiling approaches*.
v. What are the current major challenges?
  *Experimental challenges involve studying rare cell populations as well as the profiling of low-abundant targets. Furthermore, the wider adoption of rather complex workflows may require optimizations and possibilities of cost-efficient and fast sequencing turn around. Computational challenges involve data analysis and visualization due to the increasing complexity of the datasets, as well as the implementation and utilization of computational pipelines in non-expert research groups. Importantly, the increasing number of datasets generated will require the establishment of novel data integration approaches as well as the benchmarking of such computational toolkits. Additionally, standardized and open-access pipelines will be needed in order to ease the process of data mining, pre-processing, and analysing such complex datasets*.

## Supplementary information

Table EV1 Corrected

## Glossary

ACT-seq: Antibody-guided chromatin tagmentation
ATAC: Assay for transposase accessible chromatin
cDNA: Complementary DNA
ChDIP: Chromatin double immunoprecipitation
ChEC: Chromatin endogenous cleavage
ChEC-seq: Chromatin endogenous cleavage sequencing
ChIC: Chromatin immunocleavage
ChIL: Chromatin integration labeling
ChIP: Chromatin immunoprecipitation
ChIP-DIP: Chromatin immunoprecipitation done in parallel
CNV: Copy number variant
Co-ChIP: Sequential chromatin immunoprecipitation
CoTECH: Combined assay of transcriptome and enriched chromatin binding
CUTAC: Cleavage under targeted accessible chromatin
CUT&RUN: Cleavage under targets and release using nuclease
CUT&Tag: Cleavage under targets and tagmentation
Dam: DNA adenine methytransferase
DamID: DNA adenine methytransferase identification
Double ChIP: Double chromatin immunoprecipitation
ESC: Embryonic stem cell
FACS: Fluorescence-activated cell sorting
FRiP: Fraction of reads in peak
IgG: Immunoglobulin-G
LMP: Ligation-mediated PCR
MAbID: Multiplexing antibodies by barcode identification
MNase: Micrococcal nuclease
MulTI-Tag: Multiple target identification by tagmentation
mRNA: Messenger RNA
Nano-CT: Nanobody-based single-cell cleavage under targets and tagmentation
NTT-seq: Nanobody-tethered transposition followed by sequencing
pA: Protein A
pA-MNase: Protein A and micrococcal nuclease fusion protein
pA-Tn5: Protein A and Tn5 transposase fusion protein
PCR: Polymerase chain reaction
PIN*POINT: Protein position identification with nuclease tail
PIP-seq: Particle-templated instant partition sequencing
POI: Protein of interest
Pol2S5p: RNA polymerase II with phosphoserine-5
PTM: Posttranslational modification
reChIP: Sequential chromatin immunoprecipitation
scChIC-seq: Single-cell chromatin immunocleavage followed by sequencing
scChIP: Single-cell chromatin immunoprecipitation
scChIX-seq: Single-cell chromatin immunocleavage and unmixing sequencing
scCUT&Tag: Single-cell cleavage under targets and tagmentation
scCUT&Tag2for1: Single-cell cleavage under targets and tagmentation two for one
scCUT&Tag-pro: Single-cell cleavage under targets and tagmentation with cell surface proteins
scDam&T-seq: Single-cell DNA adenine methyltransferase and transcriptome sequencing
scGET-seq: Single-cell genome and epigenome by transposases sequencing
scMulti-CUT&Tag: Single-cell multi cleavage under targets and tagmentation
scPCOR-seq: Single-cell profiling of chromatin occupancy and RNAs sequencing
scRNA-seq: Single-cell RNA sequencing
seq-ChIP: Sequential chromatin immunoprecipitation
SET-seq: Same cell epigenome and transcriptome sequencing
SHARE-seq: Simultaneous high-throughput ATAC and RNA expression with sequencing
SNV: Single-nucleotide variant
sortChIC: Sort-assisted single-cell chromatin immunocleavage
SpDamID: Split DNA adenine methyltransferase identification
TAM-ChIP: Transposase-assisted chromatin multiplex immunoprecipitation
TnH: Tn5 hybrid transposase
TIRF: Total internal reflection
TSCs: Trophoblast stem cells
uCoTargetX: Ultrahigh-throughput combined tagmenting enrichment for multiple epigenetic proteins in the same cells and transcriptome
