# Publisher Correction: Emerging toolkits for decoding the co-occurrence of modified histones and chromatin proteins

**DOI:** 10.1038/s44319-024-00269-5

**Published:** 2024-10-09

**Authors:** Anne-Sophie Pepin, Robert Schneider

**Affiliations:** 1 Institute of Functional Epigenetics (IFE), Helmholtz Zentrum München, Neuherberg, Germany; 2 Faculty of Biology, Ludwig-Maximilians-Universität München, Planegg-Martinsried, Germany

## Abstract

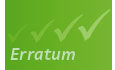

**Correction to:** *EMBO Reports* (2024) 25:3202–3220. 10.1038/s44319-024-00199-2 | Published online 2 August 2024

An issue with the selection of files related to Table EV1 was identified during the publication process.

**Table EV1 is corrected**.

## Supplementary information

Table EV1 Corrected

